# The energetic costs of escaping predation in wild, schooling white mullet (*Mugil curema*)

**DOI:** 10.1242/jeb.252375

**Published:** 2026-06-26

**Authors:** Ishani Mukherjee, James C. Liao

**Affiliations:** Department of Biology, The Whitney Laboratory for Marine Bioscience, University of Florida, Gainesville, FL 32611, USA

**Keywords:** Fish, Swimming, Escape energetics, Anti-predator behavior, Prey-predator interactions

## Abstract

Although predation is a major driver of group living across taxa and the anti-predator benefits of grouping are well established, the energetic costs experienced by groups under predation remain largely unexplored. In the current study, we use wild white mullet (*Mugil curema*) to provide real-time quantification of the energetic cost of escape in schooling fish using intermittent closed-loop respirometry. We found that small groups exposed to predators showed a 53.8% increase in their organismal metabolic rate (*Ṁ*_O_2__) compared with groups without predator exposure. When we evaluated antipredator behaviors such as escape response, group cohesion and displacement of the group centroid, we found a positive but insignificant correlation to energetic costs. We then investigated whether escape responses are socially modulated by comparing the energetic costs of escape across: (i) solitary individuals, (ii) solitary individuals with visual access to a group, and (iii) groups. We found that escape frequency and energetic costs to predation were comparable across social contexts, suggesting that escape behavior may largely reflect an intrinsic survival response rather than being strongly modulated by social context. Furthermore, we found that fish exposed to predators showed markedly reduced levels of feeding, suggesting that predation constrains energy acquisition in addition to imposing direct energetic costs. Our results provide a direct quantification of the energetic costs of escape in a schooling fish, offering new insights into the physiological trade-offs underlying collective antipredator defenses.

## Teaser

**Summary:** Wild schooling mullet incur a 54% metabolic increase when escaping predators, with energetic costs arising independently of social context and compounded by suppressed foraging.

## INTRODUCTION

Predation is one of the most powerful selective pressures shaping the behavior, physiology and life-history traits of animals across ecosystems (Lima and Dill, 1990; Dawkins and Krebs, 1979; Caro, 2005; Sih et al., 2010; Herbert-Read et al., 2017; Sabal et al., 2021; Jermacz et al., 2022). Predator pressure not only drives anti-predator strategies but also shape changes in mating strategies, foraging tactics and social organization in prey species (Sorato et al., 2012; Wilgers et al., 2014; Herbert-Read et al., 2017). One of the most widespread adaptive responses to predation is group living, which offers multiple antipredator benefits. Individuals in groups experience reduced per capita predation risk through risk dilution, collective vigilance and predator confusion (Foster and Treherne, 1981; Beauchamp et al., 2012; Beauchamp, 2013; Lehtonen and Jaatinen, 2016). Prey living in groups often exhibit striking collective behaviors when confronted with predators, including synchronized maneuvers such as rapid turns or dives (Beauchamp, 2012; Herbert-Read et al., 2015; Doran et al., 2022; Bierbach et al., 2025), and in some species, alarm signaling or mobbing behaviors that warn conspecifics or actively deter predators (Zimmermann and Curio, 1988; Radford and Ridley, 2006). At the individual level, a key component of predator avoidance across taxa is the execution of escape responses, which can determine survival during encounters (Domenici and Blake, 1997; Blumstein et al., 2016). A striking example of predator evasion is shown by saltwater schooling fish that undertake migrations spanning hundreds of miles while exposed to continuous predation from jacks, tarpons and dolphins (Furey et al., 2018; Luo et al., 2020).

Time-averaged energetic costs such as daily metabolic expenditure associated with activities such as migration, foraging and reproduction have been used to estimate overall energy budgets across species (Behrens et al., 2006; Johansen et al., 2020; Soriano-Redondo et al., 2023). While these integrative measures provide valuable insights into how animals allocate energy over extended periods, they obscure the brief, high-intensity bursts of metabolic effort that occur during rapid events such as predator evasion, leaving the immediate energetic costs of these responses largely unresolved. Quantifying these costs in real time is critical because the energetic investment in predator evasion directly constrains the aerobic capacity available for other essential activities such as migration, growth, and reproduction (Rosenfeld et al., 2015; McBride et al., 2015). In this study, we provide a quantification of the energetic costs incurred during escape in a schooling fish, offering new insight into the physiological trade-offs underlying collective antipredator defenses.

Escape events demand rapid mobilization of metabolic capacity. Because escape responses are brief but intense, they require the rapid mobilization of metabolic resources and draw directly on an individual's available physiological capacity. This capacity is commonly described in terms of aerobic scope, the difference between maximum and resting oxygen consumption. Aerobic scope defines the metabolic capacity available for functions beyond maintenance and is a key determinant of fitness (Clark et al., 2013). Elevated predation risk, through increased escape-related energy expenditure, can reduce aerobic scope and limit energy allocation to growth, reproduction, and foraging. Understanding how predation pressure interacts with aerobic capacity is therefore essential for interpreting both the immediate and longer-term energetic consequences of predator exposure. Under high or continuous predation, fish schools may also rely on their anaerobic energy reserves to escape, which is later replenished through aerobic respiration. This repayment appears as increased oxygen consumption after predation and is known as excess post-exercise oxygen consumption (EPOC) (Scarabello et al., 1992; Hancock and Gleeson, 2018).

Fish rapidly escape a threat by forming a characteristic C-bend of the body, which is actuated by Mauthner cells in the hindbrain (Eaton et al., 1977; Fetcho, 1991; Domenici and Blake, 1997). This bend is rapidly followed by body straightening that quickly accelerates the fish away from the threat. Beyond these rapid escape movements, schooling fish exhibit additional antipredator behaviors, such as increased group cohesion, reduced foraging, distancing from threats and seeking refuge. These responses are well documented in experimental studies exposing fish to live predators or predator-associated cues (Rieucau et al., 2014a,b; Nunes et al., 2019; Zanghi et al., 2023). Fish may also modify intrinsic responses to predators by gaining information on predator cues from group members (Kavaliers and Choleris, 2001; Ferrari and Chivers, 2009; Ferrari et al., 2010; Pinheiro et al., 2024; Mukherjee and Bhat, 2025). Social information can strongly influence escape dynamics, as fish in groups often initiate escape when nearby individuals react, leading to coordinated and synchronized maneuvers (Domenici and Batty, 1997; Hein et al., 2018). Interestingly, solitary fish can initiate escapes at various angles, in contrast to schooling fish, which typically escape in straight and uniform trajectories owing to the spatial constraints imposed by the school (Evans et al., 2019). Conversely, solitary fish experience isolation-induced stress and this may impact their response to a predator, sometimes decreasing escape probability or extending reaction latency (Galhardo and Oliveira, 2014). Building on these studies, we ask whether social context reshapes the energetic costs associated with escaping predators. In addition to quantifying the direct metabolic expenditures of escape, we also assess indirect costs of predation by measuring changes in foraging behavior. Together, these approaches provide a comprehensive view of how predation shapes the energy budgets of schooling fish.

White mullet are a schooling marine fish that belong to the family Mugilidae, and play a key trophic role in Florida's coastal ecosystem as an important food source for many predators (Middlemiss et al., 2018). These include dolphins (Atlantic Bottlenose, *Tursiops truncatus*), predatory birds (osprey, *Pandion haliaetus*) and a large diversity of teleost fishes (i.e. crevalle jack *Caranx hippos*; mangrove snapper *Lutjanus griseus*; bluefish *Pomatomus saltatrix*; red drum *Sciaenops ocellatus*) (Whitfield et al., 2012). White mullets are therefore under strong selective pressure in the wild to form protective schooling formations, culminating in migrations numbering in the hundreds of thousands in the summer (Thomson, 1955). During migrations, mullet schools are often exposed to continuous and repeated predation from coordinated predator groups, such as pods of dolphins (Ridgway et al., 2022). For these reasons, mullet are an ideal model for investigating the energetic costs and social context of predator evasion.

Our goals for this study were to: (1) quantify anti-predator responses and real-time energetic costs of mullet groups using intermittent closed respirometry; (2) determine whether social context modulates escape behavior and energetic costs; and (3) assess indirect energetic costs of predation by measuring feeding activity in the presence of live predators. We hypothesize that small groups of mullet responding to predator stimuli will display antipredator behaviors along with increased energetic costs. Furthermore, if escape response in schooling fish is socially driven, we expect individuals in isolation to show higher energetic costs compared with those within groups. Lastly, we hypothesize that feeding activity will decline in the presence of live predators.

## MATERIALS AND METHODS

### Fish collection and maintenance

Wild mullet (*Mugil curema* Valenciennes 1836) were collected with a cast net in January 2025 from the Matanzas River Inlet in Saint Augustine, Florida, USA. Fish (11.51±0.17 cm standard length and 25.13±0.70 g body weight; means±s.e.m.) were captured from a single school to minimize variation in age, predation history, and prior experience. After capture, fish were housed in four 588 liter rectangular tanks (63.5 cm×122 cm×76 cm) continuously supplied with fresh, UV-sterilized filtered seawater via an inflow–outflow system. Water temperature was 20±1°C, and the fish holding room was kept under a 12 h light:12 h dark cycle. Fish were fed once each day *ad libitum* with commercial food pellets (Autohime C2, Reed Mariculture, California, USA). For our experiments, we tested a total of 155 fish.

### Experiments on behavioral responses and associated energetic costs to predator stimuli

#### Experimental treatments

**Fig. 1.**
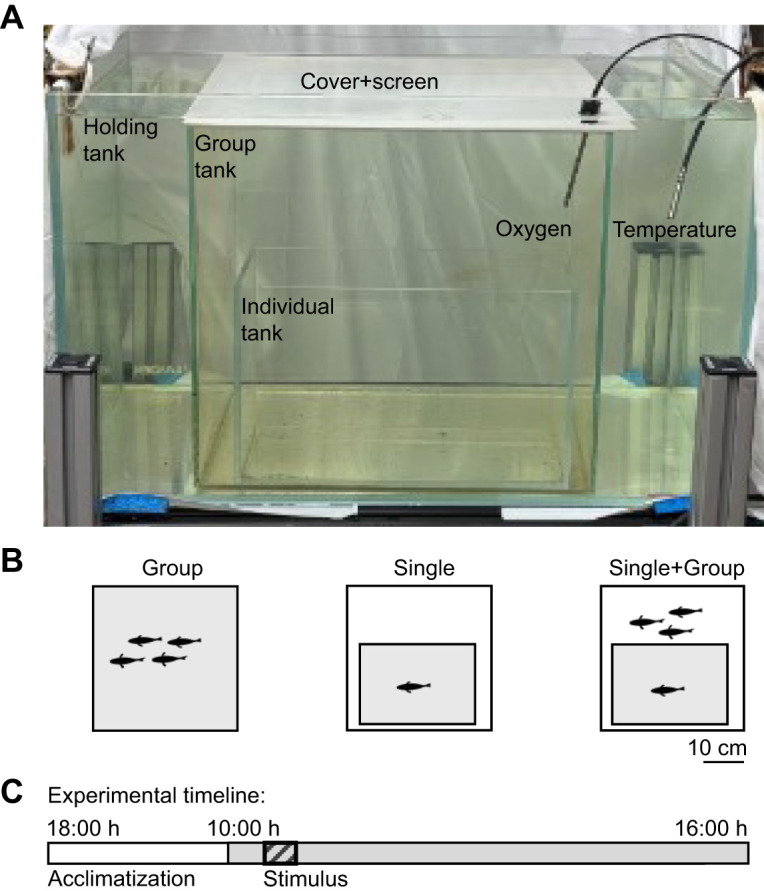
**Setup for recording oxygen consumption and behavior in individual and groups of mullet.** (A) A tank-within-a-tank setup was adopted that consisted of a outermost tank (‘holding’ tank; 41×41×61 cm, 102.5 liters), which either contained both a ‘group’ tank (36×36×36 cm, 46.65 liters) and a smaller ‘individual’ tank (29×20.5×15 cm, 8.9 liters) nested inside it, or only the group tank. Filtered sea water fully submerged the inner tank(s). The group tank was covered with a cover and screen, which enabled the projection of predator stimuli from overhead. If present, the individual tank was covered with a transparent acrylic lid. A fiberoptic oxygen sensor was inserted through a custom hole in the lid of the test tank and a temperature probe was placed directly in the holding tank. The entire setup was covered on three sides with white cloth. (B) Three social contexts tested. In the Group condition, four fish were placed together in the group tank. In the Single condition, a lone test fish was placed in a smaller individual tank nested within the group tank. In the Single+Group condition, a test fish in the individual tank could see three conspecifics swimming in the surrounding group tank. The shaded regions indicate the tank containing experimental fish. Scale bar: 10 cm. (C) Timeline for experimental trials. Protocol involved overnight acclimatization (18:00 h to 10:00 h, white bar), followed by experiments (10:00 h to 16:00 h, shaded bar). During experiments, dissolved oxygen concentration and temperature were recorded continuously in 10 min sessions. Predator treatments occurred in the third 10 min session (bar with diagonal lines), during which fish were continuously exposed to aversive visual and acoustic stimuli. In control treatments, no stimulus was presented. The schematic is not to scale.

We quantified escape responses and the associated energetic costs in mullets across different social contexts. We conducted experiments involving: (i) groups of four fish (Group), (ii) solitary individuals (Single) and (iii) solitary individuals tested within a group context (Single+Group). The Single+Group condition is a control to test potential stress arising from social isolation (Single) by placing a single fish in a transparent inner tank submerged within a larger tank housing three visibly accessible conspecifics (Fig. 1A,B). We tested ten groups of four fish. Although mullets typically form larger schools in the wild, we tested groups comprising four individuals owing to the constraints of respirometry, which required a small water volume for accurate oxygen measurements. Schooling fish primarily interact with their nearest neighbors rather than the entire group, suggesting that key social dynamics are retained even in small groups (Zampetaki et al., 2024) making laboratory studies on small groups relevant. Of the ten groups tested, we exposed five to predator stimuli and five without predator stimuli. Similarly, ten fish were tested individually, with five exposed to predator stimuli and five without. We also tested five fish that were individually exposed to predator stimuli while being in visual contact with a group. In total, we tested 55 fish for respirometry experiments. We conducted all experiments on naïve individuals. Following testing, fish were transferred to a separate tank to prevent reuse, as prior experience can influence predator responses (Ramasamy et al., 2015). We carried out experimental treatments as well as control and predator trials for each treatment in a randomized order.

#### Experimental setup

Our setup consisted of a tank-within-a-tank system to ensure that our measurement tank was free from mixing with atmospheric air, a requisite for respirometry (Lucas et al., 1993). To do this, we placed a 46.65-liter border-less cube tank (herein called the group tank, 36 cm×36 cm×36 cm) inside a 102.5 liter holding tank (41 cm×41 cm×61 cm). The system was filled with filtered seawater, ensuring that the inner tank was fully submerged. We removed air bubbles (if any) from the inner tank. To prevent water exchange between tanks, we then sealed an acrylic cover over the inner tank with a thin layer of dental wax. To carry out Single and Single+Group trials, we nested a third tank (individual tank, 8.9 liters, 29 cm×20.5 cm×15 cm). Lower tank volume in the latter treatments ensured a consistent fish mass-to-water volume ratio of ∼1:1000 across trials. Our pilot experiments revealed that these volumes ensured sufficient sensitivity to detect rapid oxygen depletion by this species and body size. In Single+Group trials, we placed one fish in the innermost tank and three others in the outer group tank. All tanks in the nested tank system were borderless and transparent, allowing visual access between the inner and outer tanks. We covered the tank-within-a-tank system on three sides with white cloth to block external visual cues, leaving one side open for video recording.

We mounted the tank system on a custom 80/20 aluminum frame (80/20 Inc., Columbia City, IN, USA). To project a looming stimulus, an overhead projector (Epson BrightLink 696Ui; 1920×1200, 60 Hz; Epson America, Los Alamitos, CA, USA) was placed above the acrylic tank lid, coated with projection paint (Goo Projector Screen Paint, Alternative Screen Solutions, MI, USA). We placed a portable waterproof speaker (EBODA Speakers, Seattle, WA, USA) adjacent to the tank system to play dolphin vocalizations. We threaded a fiber-optic optical oxygen sensor probe (Wiltrox system, Loligo Systems, Viborg, Denmark) through a fitted hole in the acrylic tank cover. We placed a temperature probe in the outer tank. We used AutoResp v3 (Loligo Systems, Viborg, Denmark) to measure oxygen saturation and temperature every second. We recorded fish movements using two Basler Pylon cameras (Basler AG, Ahrensburg, Germany; 100 frames s^−1^, 904×904 pixel resolution), one lateral and one directed at a 45 deg mirror below the tank for ventral view. We used Streampix (NorPix, Quebec, Canada) to synchronize recordings. All experiments were performed with tanks at room temperature (20±1°C).

#### Experimental protocol

Fish were transferred into the inner, experimental tank for overnight acclimation before the start of each experiment. For trials involving individuals tested within a group context, three individuals were present in the outer tank. During this period, the tank was aerated using an air stone bubbler (Marina Long Airstone, Rolf C. Hagen, MA, USA). Experiments started at 10:00 h EST the following day, when we removed the bubbler and carefully sealed the lid using a thin, leak-resistant gasket of dental wax; 10 min after sealing, we started the trials. Oxygen level and temperature were monitored inside the sealed tank every 1 s (Loligo Systems, Viborg, Denmark). If oxygen levels fell below 80%, we paused the trial, opened the tank and aerated until saturation returned to 100%. Organismal metabolic rate (*Ṁ*_O_2__) was recorded every 10 min for the duration of the experiments, which lasted 5.5 h.

In half of the trials, we introduced continuous predatory stimuli for 10 min, beginning 20 min after the start of *Ṁ*_O_2__ measurements (during the third *Ṁ*_O_2__ measurement session) (Fig. 1C). The third 10 min session was selected to apply the predatory stimulus to disentangle elevated initial *Ṁ*_O_2__ values (typically observed because of higher baseline activity earlier in the day) from the energetic demands induced by predation. To mimic the sustained natural predation faced by mullet schools, our predatory stimuli consisted of simultaneously presenting predatory dolphin vocalizations (Ridgway et al., 2022) and a visual looming stimulus. We alternately used two stimulus sizes: one expanded to cover 2.71% of the screen area and the other expanded to cover 100% of the screen. Both stimuli expanded linearly for 1 s and were played for 10 s and were alternated throughout the entire 10 min predator session to minimize habituation and maintain strong escape. We recorded both ventral and lateral video recordings during the predator stimuli.

We next measured the maximum rate of oxygen consumption for individuals (e.g. maximum aerobic metabolic rate, MMR). After the main trials, we let the fish rest for 30 min. We then subjected the fish to a standardized chase protocol (Killen et al., 2017), which involved chasing test fish with a hand net and gently touching their caudal fin for 3 min to induce exhaustive activity. Immediately after, we sealed the tank and recorded the oxygen consumption. The resulting value represented the upper physiological limit of aerobic metabolism. Following each trial, we returned fish to a separate housing tank to ensure that all individuals were subjected to the experimental procedure only once.

### Foraging behavior in the presence of live predators

We quantified foraging behavior in the presence of live predators by placing ten mullet in a 588 liter tank (63.5 cm×122 cm×76 cm) containing a native fish predator (mangrove snapper, *Lutjanus griseus*; *L*=20.28±0.57 cm; mean standard length±s.e.; Fig. 5A) contained in a cage (42 cm×36 cm×21 cm). These large tank experiments (water volume 12.6 times that of the respirometry tank) extend the scope of the study to more natural conditions. We placed a single layer of food pellets (∼4 g) onto a feeding dish at the tank bottom and recorded foraging behavior from overhead using a GoPro Hero 8 camera (120 frames s^−1^, 1080×1920 pixel resolution, GoPro, CA, USA). We tested ten groups in total. Of these, we exposed five groups to live predators (live predator treatment) and five to an empty predator cage (control). We tested 100 fish for these foraging behavior experiments. We carried out control and live predator treatments in a randomized order. After testing, fish were transferred to a separate tank to ensure no reuse. We manually counted instances of foraging, defined as when an individual swam onto the food dish and paused with its head tilted down, in a process that was blind to the presence of predators.

All experimental procedures and protocols were approved by the University of Florida Institutional Animal Care and Use Committee (IACUC ID: 202200000056).

### Data preparation

To assess group-level behavioral responses to predator stimuli, we quantified a range of anti-predator behaviors. We used lateral-view videos to manually count escape events and note the duration of each escape event. The escape duration was the sum of all escape events per trial. An escape was an all or none behavior involving a rapid fast-start swimming maneuver that allowed the fish to quickly accelerate, change direction and increase distance from the threat (Domenici and Hale, 2019). To determine whether individuals moved away from the looming stimulus (projected at the center of the tank lid) toward the bottom corners of the tank, we randomly selected six frames from lateral-view videos. Using Fiji (Schindelin et al., 2012), we measured the distance between the looming stimulus projection spot and each individual's position. We then calculated the proportion of individuals located more than 3.5 BL from the stimulus, a threshold slightly below the distance between the stimulus site and tank's bottom corners.

We tracked individuals in groups from ventral view recordings using DeepLabCut (Mathis et al., 2018) and estimated group characteristics based on the output trajectories. We calculated the total displacement of the group centroid for the first 45,000 frames to assess the movement of entire group in the presence and absence of predator stimuli for each condition. This involved first determining the group centroid (center point of polygon made by connecting position of all individuals) from individual trajectories in each frame, followed by calculating its total displacement in BL. To visualize group cohesion, we generated a heatmap plotting the relative positions of all neighboring fish with respect to each focal fish, normalized to BL. Contour lines enclosed the regions occupied by 25%, 50% and 75% of the group. To statistically compare group cohesion between treatments, we calculated mean nearest-neighbor distance (NND) or the mean distance between all four individuals across all frames.

We calculated energetic costs from measurements of the organismal metabolic rate (*Ṁ*_O_2__), based on the slope of oxygen decline over a 10 min interval. To estimate the overall behavioral responses of a group, we ranked several key metrics: (i) escape duration, (ii) displacement of centroid and (iii) mean nearest-neighbor distance (NND) low to high. A ranking summary generated a response score, where higher scores indicated stronger behavioral responses. We then quantified the correlation (Spearman's rank correlation) between *Ṁ*_O_2__ and response score.

To assess the costs experienced by mullets in the presence of live predators, we quantified foraging in presence (and absence) of a predator by manually counting the number of foraging events over a 25 min period from video recordings.

### Statistical analysis

We performed all statistical analyses using R Studio (v.2024.12.0) (r-project.org). We used unpaired Wilcoxon rank-sum tests to compare each response variable independently between predator and control conditions, in both groups and individuals. These variables included escape count, escape duration, nearest-neighbor distance (NND), group centroid displacement, the proportion of individuals located beyond 3.5 BL from the looming stimulus, and the number of feeding bites. For performing multiple comparisons (escape behavior and *Ṁ*_O_2__ across Groups, Single and Single+Group) we used Wilcoxon rank-sum tests with Bonferroni correction. Given the sample size (*n*=5 groups per condition), we employed this test as it is a non-parametric method that does not require assumptions of normality. To analyze *Ṁ*_O_2__, which was measured repeatedly over time, we used a linear mixed model using the ‘lmerTest’ package (https://CRAN.R-project.org/package=lmerTest; Kuznetsova et al., 2017). In this model, trial type (predator or control), time (0 min–5 h) and their interaction were the fixed effects and school identity was the random factor. *Post hoc* comparisons between predator and control groups at each time point were performed using Tukey's HSD test using the ‘multcomp’ package (https://CRAN.R-project.org/package=multcomp; Hothorn et al., 2008). We used Spearman's rank correlation to correlate *Ṁ*_O_2__ to response scores. All tests were two-tailed, and *P*≤0.05 was considered statistically significant. All reported values are presented as means±s.e.m.

## RESULTS

### Behavioral responses and associated energetic costs to predator stimuli

**Fig. 2.**
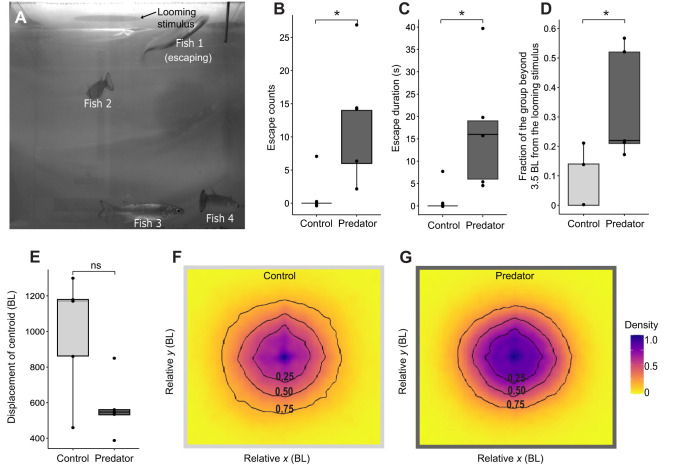
**Predator stimuli elicit changes in mullet group behavior.** (A) Representative still image from video recording showing fish responses to the looming stimulus. Fish 1 is actively escaping, whereas fishes 2–4 are not. The expanding looming stimulus is visible at the top of the frame. (B–E) Box and whisker plots showing behavior under control (light gray) and predator (dark gray) treatments. (B) Escape counts, (C) escape duration (s) and (D) fraction of the group beyond 3.5 BL from the looming stimulus were significantly greater in predator treatments compared with control treatments. (E) Displacement of the group centroid (BL) was comparable between predator and control treatments. In these box and whisker plots, each box represents the interquartile range, the line within the box indicates the median while the whiskers represent the range of the data. Each dot denotes values for behavior for a single school. Points outside the whiskers represent outliers. Five replicates per treatment were tested and comparisons were made using two-tailed unpaired Wilcoxon test. n.s. indicates no significant difference and *P<0.05, a statistically significant difference. (F,G) Heat maps depicting positions of individuals relative to a focal fish at the center (0,0) for (F) control and (G) predator treatments. Black contour lines indicate portion of group proportion (25%, 50%, 75%) around focal fish. The color bar represents the density of fish.

In the presence of predator stimuli, individuals within a group exhibited escape responses (Fig. 2A). Both the number of escape events and their durations were significantly higher in groups exposed to predator stimuli compared with controls: groups exposed to predator stimuli exhibited 12.60±0.38 escapes versus 1.40±1.20 escapes in controls (unpaired Wilcoxon test: *W*=23, *P*=0.03, Fig. 2B) and escape durations of 16.8±5.58 s compared with 1.6±1.43 s in controls (unpaired Wilcoxon test: *W*=23, *P*=0.03, Fig. 2C). Also, individuals within groups exhibited avoidance behavior by aggregating near the tank corners. A significantly greater proportion maintained a distance of at least 3.5 BL from the top center of the tank during looming stimulus presentations (0.34±0.08) compared with no-stimulus trials (0.07±0.04) (Wilcoxon test results: W=23.5, *P*=0.02; Fig. 2D).

The total displacement of the group centroid was not significantly different between predator and control groups (predator: 575.11±66.90 BL, control: 995.61±135.69 BL; Wilcoxon test results: *W*=4, *P*=0.09, Fig. 2E). Groups were more cohesive under predation risk (Fig. 2F,G), a pattern further supported by nearest-neighbor distance (NND) analyses. NND values were significantly lower during predator exposure (0.97±0.03 BL) compared with the no-exposure condition (1.12±0.04 BL; unpaired Wilcoxon test: *W*=3, *P*=0.05; Fig. S1), indicating increased group compactness as a potential anti-predator strategy.

**Fig. 3.**
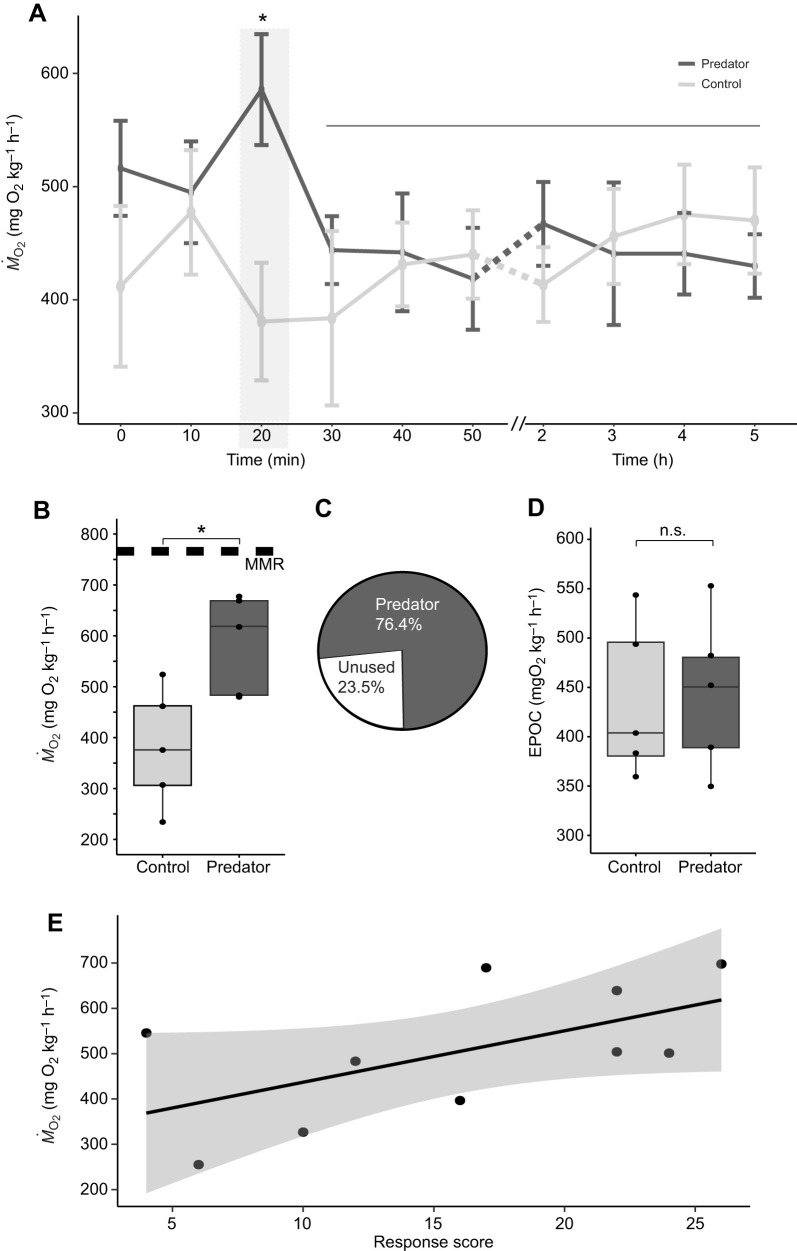
**Energetic costs of predation in groups.** (A) Temporal profile of organismal metabolic rate or *Ṁ*_O_2__ (mg O_2_ kg^−1^ h^−1^) in control (light gray) and predator (dark gray) treatments. Values represent the mean±s.e.m. of five groups. At the 20 min mark in the third session (shaded), groups in the predator treatment were exposed to predator stimuli (control groups were not exposed to predator stimuli). Horizontal line indicates time post predator exposure. (B) Box and whisker plots showing that predator-exposed fish have significantly higher *Ṁ*_O_2__ than control fish (*n*=5 schools per group, *P*<0.05). Dashed line represents the maximum metabolic rate (MMR). (C) Pie chart showing the proportion of MMR utilized during predator exposure (dark gray). (D) Mean *Ṁ*_O_2__ after predator stimuli (dark gray) is similar to control (light gray). In the box and whisker plots, each box represents the interquartile range, the line within the box indicates the median while the whiskers represent the range of the data. For B, each dot represents the *Ṁ*_O_2__ measurement for a single school, whereas in D, each dot represents the mean *Ṁ*_O_2__ across all post-exposure sessions for a given school. Points outside the whiskers represent outliers. Comparisons were performed using Tukey's HSD test. n.s. indicates no significant difference and *P<0.05 indicates a statistically significant difference. (E) Scatterplot showing a positive correlation between *Ṁ*_O_2__ and response score for all ten trials performed.

A significant treatment×time interaction (*F*=3.10, *P*=0.003), indicated that *Ṁ*_O_2__ differed between predator and control groups for time session(s) (*F*=3.09, *P<*0.01; model details in Table S1). Although *Ṁ*_O_2__ values were similar between predator and control groups before and after predator cue exposure (Fig. 3A), when predator stimuli were presented, *Ṁ*_O_2__ was significantly higher in the predator group (585.69±38.95 mg O_2_ kg^−1^ h^−1^) compared with the control group (380.79±46.69 mg O_2_ kg^−1^ h^−1^; Tukey's HSD test results for predator versus control: *t-*value=3.13, *P*<0.01, Fig. 3B). The maximum metabolic rate (MMR) was 766.58±68.04 mg O_2_ kg^−1^ h^−1^. Notably, two schools exposed to predator stimuli exhibited elevated *Ṁ*_O_2__ values (of 668.88 and 677.2 mg O_2_ kg^−1^ h^−1^) approaching the species' MMR. Overall, the energy use (mean *Ṁ*_O_2_ _of groups) under predator exposure accounted for approximately 76.4% of the total metabolic capacity. The additional energy demand due to predator presence reflects a substantial metabolic cost, leaving 23.6% of the aerobic scope unused under threat conditions (Fig. 3C).

The energetic costs to predator encounters appeared confined to the period of exposure, as no sustained elevation in *Ṁ*_O_2__ (excess post-exercise oxygen consumption or EPOC) was observed in the predator group (Fig. 3D). Specifically, the mean *Ṁ*_O_2__ from after the predator session to the end of the experiment was 448.26±32.09 mg O_2_ kg^−1^ h^−1^ and 454.12±32.67 mg O_2_ kg^−1^ h^−1^ for predator and control groups, respectively (Fig. 3D). Pairwise comparisons of *Ṁ*_O_2__ between control and predator trials across all time points, based on Tukey's HSD test, are provided in Table S2. A Spearman's rank correlation revealed a positive but non-significant association between response score and *Ṁ*_O_2__ values (ρ=0.58, *S*=69.71, *P*=0.08; Fig. 3E).

### Escape responses and associated energetic costs across social contexts

**Fig. 4.**
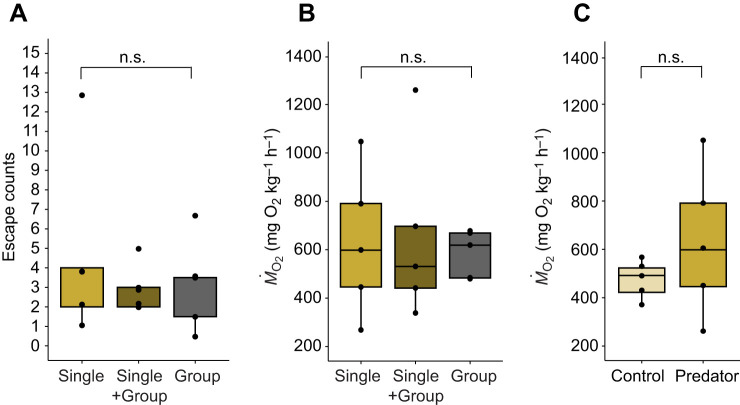
**Social context does not impact escape behavior and energetic costs imposed by predator stimuli.** Box and whisker plots showing (A) Escape counts and (B) *Ṁ*_O_2__ in predator treatments across social contexts: Single (mustard), Single+Group (dark mustard) and Group (dark gray). (C) *Ṁ*_O_2__ in a single fish across control (light mustard) and predator (mustard) treatments. Each box represents the interquartile range, the line within the box indicates the median while the whiskers represent the range of the data. Each dot denotes escape count or *Ṁ*_O_2__ value for a single school. Points outside the whiskers represent outliers. Five replicates per treatment were tested and comparisons were made using two-tailed unpaired Wilcoxon test with Bonferroni correction. n.s. indicates no significant difference across treatments.

Individuals in a group did not always escape in synchrony. Among the 28 escape events observed across the five predator-exposed groups, all four individuals escaped together in only 9 out of 28 events. In the remaining events (19 out of 28), partial group escapes were observed (Fig. S2). We found comparable escape count per individuals among Single (4.80±1.90 escapes), Single+Group (3.00±0.48 escapes) and Group (3.15±0.95 escapes; Unpaired Wilcoxon test with Bonferroni correction results: Single vs. Group: *W*=9, p=1; Single+Group vs. Single: *W*=11, p=1; Single+Group vs. Group: *W*=12, p=1; Fig. 4A). Similarly, the *Ṁ*_O_2__ during exposure to predator stimuli was comparable across the individuals: Single (630.30±120.77 mg O_2_ kg^−1^ h^−1^), Single+Group (653.32±145.42 88 mg O_2_ kg^−1^ h^−1^) and Group (585.68±38.94 mg O_2_ kg^−1^ h^−1^) (Wilcoxon test with Bonferroni correction results: Single vs. Group: *W*=13, p=1; Single+Group vs. Single: *W*=12, p=1; Single+Group vs. Group: *W*=12, p=1; Fig. 4B). *Ṁ*_O_2__ levels were elevated even in the absence of predator stimuli for the Single treatment (472.91±32.55 mg O_2_ kg^−1^ h^−1^) and did not differ significantly from levels recorded during predator exposure (630.30±120.77 mg O_2_ kg^−1^ h^−1^; Wilcoxon rank-sum test: *W*=17, *P*=0.42, Fig. 4C).

### Feeding suppression by live predators

**Fig. 5.**
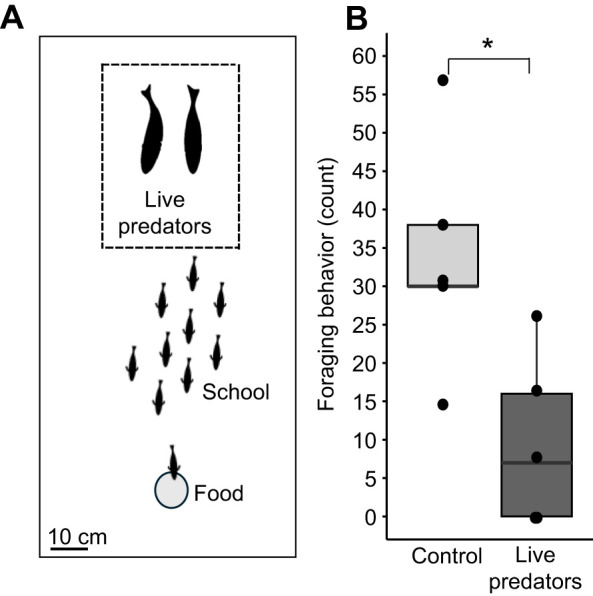
**Indirect energetic costs in the presence of a predator.** (A) Experimental setup showing a predator cage (comprising two live predators), a school of test fish (10 mullet) and a food source. Scale bar: 10 cm. (B) Foraging behavior (counts) depicted using box and whisker plots in which each box represents the interquartile range, the line within the box indicates the median whereas the whiskers represent the range of the data. Each dot denotes foraging behavior count for a single school, dots beyond the whiskers are outliers. Foraging behavior was significantly lower in live predator-exposed groups than in controls (*n*=5 schools per group, *P<0.05). The comparisons were made using two-tailed unpaired Wilcoxon tests.

To account for indirect energetic costs of predation risk, we exposed mullet to live predators (e.g. mangrove snapper; Fig. 5A). Exposure to snappers led to a ∼71% suppression in foraging activity (9.80±4.47 bites) relative to control groups (33.80±6.25 bites; Wilcoxon rank-sum test: *W*=2, *P*=0.03; Fig. 5B).

## DISCUSSION

Our study measures both the direct, energetic and indirect, behavioral costs of a schooling fish escaping from a predator. Groups of wild mullet quickly distance themselves from predator stimuli by performing rapid escapes with heightened school cohesion. Interestingly, we found that escape energetics and behavior were similar for individual fish regardless of whether they were solitary or with a group. We also observed that groups exposed to live predators suppressed foraging activity. By quantifying the metabolic cost of anti-predator behavior in schooling fish, this study considers physiological, behavioral and ecological perspectives to better understand the nature of predator–prey interactions.

### Behavioral responses and associated energetic costs to predator stimuli

A group of mullet, as well as individuals within the group, showed pronounced anti-predator behaviors when exposed to predator cues. Individuals within groups escaped to continuous looming stimuli and auditory cues, similar to other fish species (Lefrançois et al., 2005; Marras and Domenici, 2013; Domenici and Hale, 2019; Rodriguez-Pinto et al., 2024), and positioned themselves away from the stimulus source. Similar spatial avoidance to projected looming stimuli has been found in natural marine habitats, where coral reef fishes adjust their escape trajectories to steer away from threats and swim towards shelter (Hein et al., 2018). We found no significant change in centroid displacement of groups in the presence of a predator (a metric that reflects overall group movement; Herbert-Read et al., 2013; Jolles et al., 2017), which may suggest that centroid-based metrics provide only an estimate of school movement, and fails to capture critical aspects of antipredator behavior such as fine-scale individual escape maneuvers. For instance, if individuals escape in opposite directions, centroid displacement will resemble the lack of response characterizing the pre-predator state. As a result, centroid-based measures may overlook within-group movement variation. This limitation could account for the lack of difference between control and predator-exposed schools. Another possibility for the comparable centroid displacements is that the limited sample size reduced our ability to detect subtle differences between control and predator-exposed schools. Under predator stimuli, heightened cohesion may not only confuse predators and dilute individual risk but also enhance the speed and precision of collective escape responses (Foster and Treherne, 1981; Lima, 1995; Herbert-Read et al., 2017). Similar to our study, other schooling species such as golden shiners (*Notemigonus crysoleucas*), guppies (*Poecilia reticulata*), fathead minnows (*Pimephales promelas*) and zebrafish (*Danio rerio*) also show greater cohesion upon exposure to predation (Johannes, 1993; Chivers et al., 1995; Herbert-Read et al., 2017; Ioannou et al., 2017; Mukherjee and Bhat, 2024).

Our study demonstrates that the anti-predator responses deployed by groups under predation risk come at a substantial energetic cost (e.g. a 53.8% increase in oxygen uptake). Thus, our findings highlight that although these responses may reduce the likelihood of predation, they do so at a significant energetic cost for both individuals and groups. The resulting increase in physiological demand may constrain individual energy budgets, with consequences for growth, reproduction and longer-term fitness. Interestingly, oxygen uptake was 7.06% higher during the pre-exposure session (in both predator and control trials) compared with the later control sessions. However, this difference was not statistically significant (Fig. S3) and should therefore be interpreted with caution. The observed pattern may reflect variation in activity levels given that fish are often more active in the morning (Helfman, 1981; Bosiger and McCormick, 2014), leading to elevated oxygen consumption, or alternatively, could arise from mild stress associated with tank sealing prior to the experiment and the subsequent brief secondary acclimation period. Owing to the prevalence of a slightly higher (although statistically insignificant) oxygen consumption in the morning, we compared our findings with a control performed on a separate group rather than to pre-predator measurements from the same group.

While a predatory stimulus substantially increased oxygen consumption in mullet, it remained below their maximal metabolic rate (MMR), suggesting that mullet can tolerate prolonged predation. MMR and aerobic scope are related to lifestyle across fish species, with sedentary species showing low values and more athletic species displaying relatively high values (Norin and Clark, 2016; Norin and Metcalfe, 2019; Jermacz et al., 2020; Fu et al., 2022). This framework is consistent with our results: migratory mullet possess high MMR and aerobic scope, reflecting their active lifestyle. Likewise, our results indicate no significant energetic carry-over effects of predation: the *Ṁ*_O_2__ measured from the end of the predator session to the end of the experiment did not differ between predator and control groups. This result is contrary to what has been found in other marine fishes. For instance, striped surfperch (*Embiotoca lateralis*) exhibit elevated EPOC (excess post-exercise oxygen consumption) following prolonged swimming under flow (Cordero et al., 2019). Atlantic salmon (*Salmo salar*) show similar post-exercise costs after exhaustive chase trials (Zhang et al., 2018). Closely related, golden gray mullet (*Chelon auratus*) exhibit energetic costs after predator attacks, with faster-responding individuals showing higher EPOC, highlighting a trade-off between vigilance and post-exercise metabolic cost (Killen et al., 2015). The elevated EPOC in previous studies probably reflects the use of a mechanical stimulus (e.g. dropping a PVC pipe), designed to mimic a sudden predator strike. In contrast, our aversive stimuli were patterned after the chronic presence of predators, as might be perceived by a fish school under repeated, sustained attack. Thus, the absence of carry over effects, together with the finding that *Ṁ*_O_2__ remained below MMR during predation, is consistent with the ecology of wild mullets. Migrating mullet schools face intense predation risk (Richards and Castagna, 1976) and may have evolved to withstand such challenges. Their ability to maintain predation costs below MMR and within aerobic scope, without carry-over effects, may be particularly advantageous in coastal habitats where predator encounters are frequent and unpredictable (Mosman et al., 2023).

Although the relationship between response score and *Ṁ*_O_2__ was positive, it was not statistically significant. This is likely because antipredator responses are multidimensional, encompassing behaviors such as rapid escapes, cohesion, and subtle postural adjustments, many of which may not be fully captured in our study and within the response score. Furthermore, individuals are likely to exhibit consistent behavioral differences that are closely linked to their internal state (Sih et al., 2015), leading to variation in both antipredator responses and the associated oxygen consumption. Experimental evidence further supports this, showing that state–behavior feedback loops, such as those between boldness and food intake, can shape escape performance and generate trade-offs in antipredator responses (Planas-Sitjà and Ioannou, 2025). Additionally, although our findings reveal consistent patterns, the relatively small sample size may have limited our ability to detect subtle effects, particularly in the relationship between behavioral responses and energetic costs. These considerations suggest that although stronger antipredator responses tend to be associated with higher energetic costs, fully resolving this relationship may require finer-scale behavioral classification and the examination of predator induced changes in physiology in greater details. Similar to our finding, several other fish species such as hammerhead sharks (*Sphyrna lewini*) and southern catfish (*Silurus meridionalis*) show a positive correlation between activity and *Ṁ*_O_2__ (Gleiss et al., 2010; Zhang et al., 2010).

### Escape responses and associated energetic costs across social contexts

We expected that schooling would reduce energetic costs owing to hydrodynamic wake recapture (Liao, 2022) and social buffering of stress (Culbert et al., 2019). However, we did not detect clear differences in escape frequency or energetic costs between individuals with and without visual access to a group under our experimental conditions. Our study thus suggests that although grouping may reduce the likelihood of successful predator attacks, grouping does not appear to confer energetic advantages during predator evasion. This discrepancy likely arises because mullets did not always exhibit coordinated escape maneuvers. Nonetheless, some evidence of behavioral coupling does exist: Fig. S1 shows that frequency of all four fish responding together was much higher than instances of three fish responding. This pattern indicates a tendency for individuals to match the responses of others-once most group members initiate an escape, the remaining individuals may do so as well. At the same time, not all responses were fully coordinated, and individuals sometimes fled asynchronously or in different directions, which could disrupt the spatial organization required to generate hydrodynamic benefits (Weihs, 1975; Fish and Lauder, 2006). The presence of asynchrony during escapes may reflect existence of individual in addition to collective decision-making under threat. Given that schooling can provide hydrodynamic advantages under flow (Zhang and Lauder, 2024), we speculate that the presence of water flow may promote more coordinated escape responses among individuals, thereby conferring energetic benefits to solitary fish in ways that were not observed for our fish in still water.

Note that solitary individuals exhibited elevated *Ṁ*_O_2__ even in the absence of predator stimuli, which may reflect a level of stress associated with isolation. Higher energetic costs and stress due to isolation have also been previously reported across various fish species (Galhardo and Oliveira, 2014; Forsatkar et al., 2017; Xu et al., 2024).

### Costs faced by schools facing live predators

The costs of being exposed to predators are not restricted to the immediate energetic demands of escape but also include indirect effects in the form of reduced food uptake. Several fish species decrease their feeding activity in the presence of predators (Des Roches et al., 2022; Shapiro Goldberg et al., 2021; Mukherjee and Bhat, 2025; Ling et al., 2019). Reduced food intake can limit the energy available for sustaining high metabolic performance and growth (Auer et al., 2015; Ling et al., 2019), thereby constraining long-term fitness. Although our study quantifies the direct energetic costs associated with heightened oxygen demand during escape behaviors, it also reveals indirect costs arising from behavioral trade-offs such as reduced foraging.

We propose several directions for future work. Measuring latency to escape in response to looming stimuli could provide insights into the responsiveness of wild mullet and may serve as an indirect indicator of the perceived strength of chronic predator stimuli. Also, introducing hydrodynamic flow could test whether predator-induced energetic costs are exacerbated under stress. Finally, investigating neurophysiological correlates, for example through lateral line ablation, could help elucidate the sensory contributions to escape energetics. These approaches would deepen our understanding of how social and environmental factors shape the metabolic consequences of anti-predator behavior in schooling fish. Lastly, investigations quantifying escape behaviors in the wild would be invaluable. In nature, mullets escape predators by fleeing over greater distances and also display behaviors such as leaping out of the water (Peterson, 1976; Whitfield et al., 2012). The confined boundaries of laboratory tanks prohibit a comprehensive understanding of the strategies and costs of predator evasion.

By directly linking escape behavior to metabolic expenditure, our study shows that predator encounters impose substantial yet physiologically moderate energetic costs, and that these costs arise independently of social context. Although escape responses in the wild are likely influenced by group size and dynamic environmental conditions (Rieucau et al., 2014a,b), we argue that our experiments on small groups under controlled laboratory conditions capture key behavioral and energetic patterns observed in nature, even if they cannot be directly scaled to populations in the wild. The additional suppression of feeding under predation further underscores that predators influence not only the immediate energetic demands of escape, but also the longer-term balance between energy intake and expenditure. More broadly, our work provides a rare empirical window into the energetic underpinnings of collective behavior and highlights how both direct and indirect metabolic pressures may shape the evolution of sociality, foraging strategies, and risk management across animal taxa.

## Supplementary Material

10.1242/jexbio.252375_sup1 Supplementary information
