# Supplementary material for: The energetic costs of escaping predation in wild, schooling white mullet (Mugil curema)
Source: J Exp Biol. 2026 Jun 26;229(12):jeb252375. doi: 10.1242/jeb.252375 (PMC13354954; doi:10.1242/jeb.252375)
Supplement: Supplementary information [file jexbio-229-252375-s1.pdf]

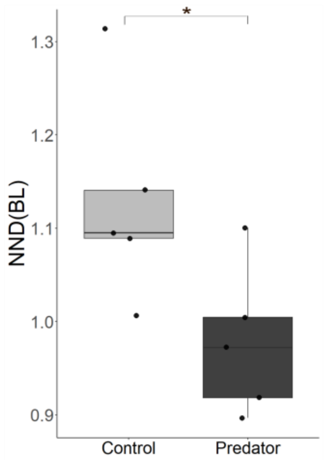

Fig. S1. Lower nearest neighbor distance in predation in groups. Box and whisker plots showing predator-exposed fish have significantly reduced NND (dark gray) than Control fish (light gray, $p < 0.05$). Each dot denotes escape the NND value for a single school. * indicates a statistically significant difference.

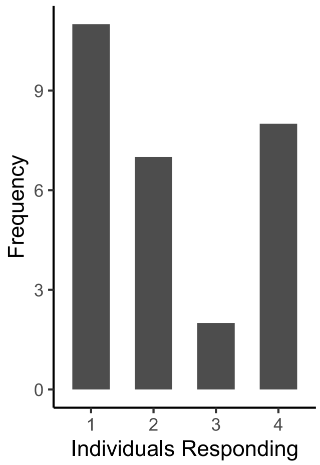

Fig. S2. Bar plot showing the frequency of escape responses by one to four individuals following predator stimuli. Simultaneous escape by all four individuals occurs relatively infrequently compared to partial group responses (one to three individuals escaping), indicating that escape behavior is not fully synchronized.

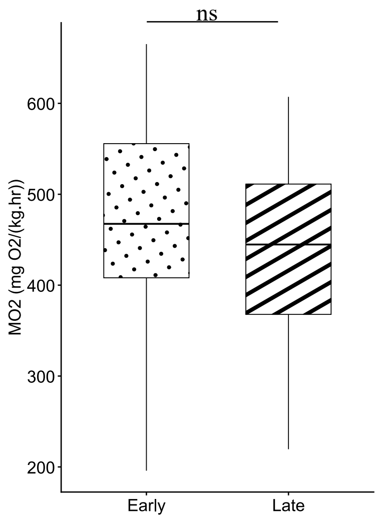

Figure S3. Comparable energetic costs during early and late MO_2 measurement sessions. Box and whisker plots showing comparable energetic costs during early and late MO2 measurement sessions ($p < 0.05$). ns indicates no statistically significant difference.

Table S1. Results of the LMM for predicting the effect of time and treatment on MO_2

Model: MO_2~Treatment*Time+(1| School ID)

	Sum Sq	Mean Sq	Num df	Den Df	f value	p
Treatment	2611	2610.8	1	1.80	0.58	0.46
Time	54895	6099.4	9	70.04	1.35	0.22
**Treatment*Time**	**125127**	**13903.0**	**9**	**70.04**	**3.09**	**<0.01**

Journal of Experimental Biology • Supplementary information

Table S2. Tukey's HSD test results for comparisons between Predator and Control groups at each time point:

MO2 measurement session	Tukey's HSD test results for comparisons of Control vs. Predator				
	Estimate	SE	df	t ratio	p value
0min	104.3	65.4	19.8	1.594	0.12
10min	17.7	65.4	19.8	0.27	0.78
**20min***	**204.9**	**65.4**	**19.8**	**3.13**	**0.005**
30min	71.5	67.3	21.8	1.06	0.29
40min	10.7	65.4	19.8	0.16	0.87
50min	-29.7	67.3	21.8	-0.44	0.66
2hr	53.7	65.4	19.8	0.82	0.42
3hr	36	65.4	19.8	0.55	0.58
4hr	-34.8	65.4	19.8	0.53	0.60
5hr	-40.2	65.4	19.8	-0.61	0.54

*Predator cues presented in this session in 50% schools
